# Genetic engineering to enhance microalgal-based produced water treatment with emphasis on CRISPR/Cas9: A review

**DOI:** 10.3389/fbioe.2022.1104914

**Published:** 2023-01-13

**Authors:** Alaa Hassanien, Imen Saadaoui, Kira Schipper, Sara Al-Marri, Tasneem Dalgamouni, Mustapha Aouida, Suhur Saeed, Hareb M. Al-Jabri

**Affiliations:** ^1^ Algal Technologies Program, Center for Sustainable Development, College of Arts and Sciences, Qatar University, Doha, Qatar; ^2^ Biological and environmental Sciences Department, College of Arts and Sciences, Qatar University, Doha, Qatar; ^3^ ExxonMobil Research Qatar (EMRQ), Doha, Qatar; ^4^ Division of Biological and Biomedical Sciences, Qatar Foundation, College of Health and Life Sciences, Education City, Hamad Bin Khalifa University, Doha, Qatar

**Keywords:** bioremediation, CRISPR/cas9, genetic engineering, microalgae, produced wastewater

## Abstract

In recent years, the increased demand for and regional variability of available water resources, along with sustainable water supply planning, have driven interest in the reuse of produced water. Reusing produced water can provide important economic, social, and environmental benefits, particularly in water-scarce regions. Therefore, efficient wastewater treatment is a crucial step prior to reuse to meet the requirements for use within the oil and gas industry or by external users. Bioremediation using microalgae has received increased interest as a method for produced water treatment for removing not only major contaminants such as nitrogen and phosphorus, but also heavy metals and hydrocarbons. Some research publications reported nearly 100% removal of total hydrocarbons, total nitrogen, ammonium nitrogen, and iron when using microalgae to treat produced water. Enhancing microalgal removal efficiency as well as growth rate, in the presence of such relevant contaminants is of great interest to many industries to further optimize the process. One novel approach to further enhancing algal capabilities and phytoremediation of wastewater is genetic modification. A comprehensive description of using genetically engineered microalgae for wastewater bioremediation is discussed in this review. This article also reviews random and targeted mutations as a method to alter microalgal traits to produce strains capable of tolerating various stressors related to wastewater. Other methods of genetic engineering are discussed, with sympathy for CRISPR/Cas9 technology. This is accompanied by the opportunities, as well as the challenges of using genetically engineered microalgae for this purpose.

## 1 Introduction

Microalgae are photosynthetic microscopic organisms, either prokaryotic or eukaryotic that could live in all bodies of water and utilize sunlight and carbon dioxide (CO_2_) as their sole energy and carbon sources to produce organic compounds *via* photosynthesis (Zhu et al., 2016; Deviram et al., 2020). This, together with their fast growth rates, and ability to produce high value metabolites of interest for many industrial applications, make microalgae an attractive subject for researchers in the field of sustainable production (Saadaoui et al., 2019; Arias et al., 2020; Rasheed et al., 2020; Saadaoui et al., 2019; Veiga et al., 2020; Saadaoui et al., 2019; Varaprasad et al., 2021; Bounnit et al., 2022; Bello et al., 2022). Furthermore, microalgae have also been found to be able to efficiently remove contaminants from various types of wastewater effluents, including pharmaceutical (Singh et al., 2020), agricultural (Leite et al., 2019), and industrial (Al-Jabri et al., 2021). In the tertiary treatment stage, they can eliminate macro-nutrients from the water mainly nitrogen and phosphorus, as well as heavy metals (Molazadeh et al., 2019). Even in particularly contaminated wastewaters, such as produced water from the oil and gas industry, microalgae have demonstrated the ability to degrade specific components as a treatment step toward clean water (Graham et al., 2017; Rahman et al., 2020; Alsarayreh et al., 2022). Overall, cultivation of microalgae on different types of wastewaters is considered a sustainable technology for bioremediation due to its capacity to thrive by consuming present contaminants (Mehariya et al., 2021). Non-etheless, due to immature technologies, instabilities of wastewater components, and required improvements of bioremediation efficiencies, microalgae are not yet widely applied for wastewater treatment (Feng et al., 2020; Li et al., 2022).

Various methods can be applied to enhance the bioremediation efficiencies of microalgae, including advanced cultivation systems, consortiums, and genetic modification. Microalgal cells are transformable; their genomes can be redesigned to include a desired feature by employing the proper delivery system to introduce DNA for transformation (Gimpel et al., 2015). Transformation of different microalgal species using multiple genetic engineering tools has been successfully applied to enhance metabolite production for biofuels (Radakovits et al., 2010) as well as for other products (Ibuot et al., 2017; Rahman et al., 2020). Genetic engineering is a powerful tool to enhance the ability of many microorganisms’ to bioremediate wastewater. For example (Huang et al., 2015), inserted the arsenite S-adenosylmethionine methyltransferase gene obtained from the red microalgae *Cyanidioschyzon merolae* into *Bacillus subtilis*. The transformed *Bacillus* was able to methylate the inorganic arsenic. Such studies provide proof for concept of using transformed microbes for treating different kinds of contaminants.

This review provides the status of microalgal-based bioremediation of produced water, as well as the most recent microalgal applicable genetic engineering tools: zinc finger nucleases (ZFNs), Transcription activator-like effector nucleases (TALENs), and clustered regularly interspaced short palindromic repeats (CRISPR/Cas9). Previous reviews of this field have particularly focused on multiple wastewater treatment such as agricultural or municipal, or on genetic engineering as a tool to enhance efficiency the efficiency of microalgae to serve different purposes. This review is the first of its kind to develop a critical overview and explore about the importance of genetic engineering in enhancing algae phytoremediation efficiency. Combining the two topics allows us a forward look at genetic engineering strategies to enhance microalgal efficiency in phytoremediation of produced wastewater.

## 2 Microalgae: Promising alternative for produced water bioremediation

Produced water (PW) is the highest volume of liquid waste generated and discharged during the production of oil and gas. The worldwide volume of produced water generated is approximately 1.3 times that of hydrocarbon production (Gray, 2020). PW varies in composition and volume from one formation to another, and predominant constituents include total dissolved solids (TDS), such as natural salts and minerals, as well as dissolved and volatile organic compounds, oil and grease, heavy metals, dissolved gases, bacteria, naturally occurring radioactive materials (NORM, radionuclides such as radium), and the additives used in hydrocarbon production (Al-Ghouti et al., 2019). In recent years, the increased demand for and regional variability of available water resources, along with sustainable water supply planning, have driven interest in the reuse of produced water. As freshwater supplies become scarcer, produced water can be a crucial source of water after suitable treatment. There has been an increased focus on reclaiming, reusing, and recycling water that is usually wasted to meet the communities’ needs for freshwater sources (Gray, 2020).

In recent times, a greater focus has appeared on using biological systems, including microalgae, for treating produced wastewater effluents (Graham et al., 2017; Rahman et al., 2020; Alsarayreh et al., 2022). Application of microalgae in wastewater treatment even shows competitive advantages over other treatment methods to improve water quality, due to its high treatment efficiency as well as carbon capture and biomass valorization opportunities (Molazadeh et al., 2019; Leng et al., 2020; Alsarayreh et al., 2022).

It is not surprising that algae have a high potential for PW treatment. For decades, researchers have studied their general wastewater treatment capabilities, optimizing their treatment efficiencies and growth on different types of wastewaters. Salgueiro et al. (2016) for example demonstrated that *Chlorella vulgaris* was able to remove 99.2% of phosphorus from artificial wastewater containing glucose, ammonium chloride, urea, monopotassium phosphate and reduce the chemical oxygen demand (COD) by 71.1%. Likewise (El-Kassas and Mohamed, 2014), showed that Chlorella vulgaris was able to treat textile wastewater, with COD and color removal percentages of 69.9% and 76.32%, respectively. Furthermore (Wang et al., 2019), investigated the use of the marine diatom *Phaeodactylum tricornutum* to treat municipal wastewater mixed with seawater. The results revealed that the diatom was able to remove up to 89.9% of COD, 86.7% of Total Nitrogen (TN), 84.2% of ammonium, and 97.0% of total phosphorus. Moreover, the strains were able to produce a high amount of lipids, which could potentially be applied for biodiesel production (Jayakumar et al., 2021). Produced water, on the other hand, can be more challenging, due to potential toxicity and the presence of heavy metals, hydrocarbons, surfactants, and anti-corrosives. Non-etheless, various studies have shown that bioremediation of PW using microalgae has shown a high potential. For example (Das et al., 2019), demonstrated the ability of Chlorella sp. (QUCCCM10) to bioremediate pretreated PW, after pH adjustment and removal of suspended matter. The microalga was found to be able to reduce the concentration of various elements, such as arsenic, cadmium, iron, nickel, and potassium, as well as remove 92% and 94.2% of TN and Total Organic Carbon (TOC), respectively. In a more recent study (Rahman et al., 2021), showed that *Galdieria sulphuraria* was able to grow in PW concentrations of up to 50%, with biomass productivities of up to .72 g L^−1^·d^−1^, and 99.6% and 74.2% nitrogen and phosphorus removal rates, respectively, without the addition of extra micronutrients. Similarly (Ammar et al., 2018), inoculated two marine microalgae species, Nannochloropsis oculata and Isochrysis galbana, with different concentrations of PW obtained from an oil field located in Iraq. Although higher PW loadings were found to have a negative impact on biomass productivities; successive adaptation biomass yields of .31 g L^−1^ were still achieved for both strains at 50% PW loading. Optimal contaminant removal however occurred at lower PW loadings of 10% and 25%, at which N*annochloropsis oculata* was able to remove up to 89% and 81% oil content and 90% and 72% COD, respectively.

The ability of microalgae to remove pollutants from wastewater is due to different mechanisms they can perform. First, microalgae have the ability to take advantage of mixotrophic modes of nutrition. Which means it can switch their metabolism from using only CO_2_ as a carbon source to using organic matter based on its availability in the growth medium (Alalawy et al., 2019). (Devi et al., 2022) cultivated *Scenedesmus* sp. DDVG strain in municipal wastewater under mixotrophic condition to test the strain availability to survive and remove major contaminants form the water. The results showed that *Scenedesmus* efficiently removed ≈75% of COD and ≈100% of total nitrogen with a biomass of nearly 3.4 gL^−1^ after 10 days of cultivation.

Microalgal removal of organic compounds from the wastewater can be credited to biodegradation and biosorption processes. Biodegradation is known to be the most effective method by which microalgae use different enzymes to eliminate organic micropollutants from the aqueous environment (Nguyen et al., 2021). Biosorption is defined as the physical-chemical process in which substances are removed from solution using biological matter. Due to the negative charge of the cell wall in microalgae, cationic pollutants can efficiently adhere to the surface. However, it is less efficient in term of pollutants removal compared to biodegradation (Nguyen et al., 2021). Biodegradation and biosorption of different contaminants including organic compounds are extensively studied by Pathak et al. (2018).

Microalgae have enormous potential not only for bioremediation of produced water, but also for biomass reutilization in a variety of applications. Algae cultivated in wastewater are rich source of primary and secondary metabolites that could benefit in producing many valuable bioproducts such as biofuels, biofertilizers, and feed supplements (Shahid et al., 2020). Combining algae-based wastewater treatment with producing high-value compounds will greatly reduce the economic cost of the process. For instance (Japar et al., 2021), successfully increased the biomass production and lipid content of *Chlorella vulgaris* and *Chlorella sorokiniana UKM3* grown in industrial wastewater by acclimatization process. . Since wastewater can be used as a sustainable growth medium, a variety of wastewater types have been proposed to increase algal biomass (Srimongkol et al., 2022). In this context, biomass generated from produced water treatment can be a promising source for valuable metabolite production for numerous applications. In Table 1, more detailed information is shown on these and other recent studies investigating algal-mediated PW treatment, including the different strains, experimental and cultivation conditions applied.

**TABLE 1:** Selected recent literature on algal bioremediation of PW.

	Strain	Wastewater type	Cultivation conditions	Highest biomass yield (g·L^−1^)	Pollutants removed	Highest removal efficiency (%)	Reference
*1*	*Chlorella sp*	produced water from a Qatari local petroleum company	In a temperature-controlled room, a glass bottle was agitated with compressed air and illuminated with an light intensity of 600 µmol photons·m^−2^·s^−1^	1.72	TOC	73	Das et al. (2019)
					total Nitrogen	92	
*2*	*Chlorella vulgaris*	Produced water from an oil and gas facility in United States.	Tissue Culture Roller Drum Apparatus inside an incubator with a constant level of CO_2_ of 2%–3% (v/v), a temperature of 28°C with 16:8 h light:dark cycle and an illumination of ∼4000 lux	3.1 ± 0.5	total Nitrogen	100	Rahman et al. (2021)
					phosphorus	≈74.2	
*3*	*Chlorella vulgaris*	PW from dumping site generated by oil wells in Colombia	fluorescent light at an irradiance of 36.8 ± 4.2 μmol photons m^−2^ s^−1^ at the surface of the culture medium, temperature at 20 °C and permanent aeration supplied by a blower	—	Total hydrocarbons	≈100	Calderón-delgado et al. (2019)
*4*	*Chlorella pyrenoidosa*	PW from oilfield in Algeria	outdoor, under sunlight radiation, using an open system sited in the desert area in the winter season. The temperatures fluctuated from 26 to 31°C during the day	1.15	COD	89.67%	Rahmani et al. (2022)
					Ammonium nitrogen	100%	
					total Nitrogen	57.14%,	
					total Phosphorus	75.51%	
					Copper	73.39	
					Lead	72.80	
					Cadmium	48.42	
*5*	*Nannochloropsis oculata*	Produced water from oil field in Iraq	Florescence light (2000 lux) at and a light photoperiod of 18:6 h light:dark, 25°C±1°C, continuous filtered air at a constant flow rate *via* two aquarium air pumps	1.13	Oil	66.5	Ammar et al. (2018)
					COD	54	
*6*	*Nannochloropsis oculata*	Produced water from a TOTAL operating site in France	14/10 h light/dark periods, by LED lamps, temperature at (21 ± 1°C), autotrophic conditions with air. CO_2_ was added in pulse, 5 s each 20–40 min and pH between 7.5 and 9	—	Ammonium nitrogen	≈100	Parsy et al. (2020)
					COD	70	
					Iron	100	
*7*	*Nannochloropsis oculata*	Produced water from an oil field in Brazil	Aerated photobioreactors (3 L min^−1^), cold white LED lamps with light intensity of 57 μmol m^−2^·s^−1^, photoperiod of 12:12 h. Temperature controlled at 21°C±.9°C. The pH was fixed at 7	—	PAHs	94	Marques et al. (2021)
					NAP	96	
					APT	95	
					FLU	91	
					PHE	83	
					BbF	95	
					DA	90	
					BaP	95	
					Iron	96.80	
*8*	*Galdieria sulphuraria*	Produced water from an oil and gas facility in United States.	Tissue Culture Roller Drum Apparatus inside an incubator CO_2_ level was kept constant at 2%–3% (v/v), temperature 42°C with 24 h of continuous illumination (∼4000 lux)	5.12 ± .28	total Nitrogen	≈100	Rahman et al. (2021)
*9*	*Isochrysis galbana*	Produced water from oil field in Iraq	Florescence light (2000 lux), and a photoperiod of 18:6 h light: dark, 25°C±1°C, continuous filtered air at a constant flow rate *via* two aquarium air pumps	1.01	Oil	68	Ammar et al. (2018)
					COD	56	
*10*	*Dunaliella tertiolecta*	Produced water from an oil production facility in the Permian Basin of southeast New Mexico	Temperature controlled at 24°C in a growth chamber with fluorescent illumination of 100 μmol photons m^−2^ s^−1^. agitation was set at at 140 rpm with a 16-h light/8-h dark cycle	≈0.3	nitrate	≈99.6	Hopkins et al. (2019)
					Phosphate	≈99.6	

PAHs, polycyclic aromatic hydrocarbons; NAP, naphthalene; AP, acenaphthylene; FLU, fluorene; PHE, phenanthrene; BbF, benzo(b)fluoranthene; DA, dibenzo (a, h) anthracene; BaP, benzo(a)pyrene.

To summarize, utilizing microalgae for PW bioremediation can be advantageous and efficient owing to i) its capability of removing heavy metals, hydrocarbons, and other pollutants, ii) its ability to produce high-value bioproducts such as biofuels (Cho et al., 2011), iii) its ability to reduce the need for fresh water and nutrients for algae cultivation (Rahman et al., 2020; Ahmad et al., 2021) and iv) its potential for carbon capture and utilization (CCU) from industrial point-sources (Kalra et al., 2021). Non-etheless, some obstacles and complications still arise when applying this technology, which will need to be mitigated for it to be applied on a large scale. Such concerns include the requirement for long residence times for efficient contaminant removal, the risk of contamination, the existence of contaminants that may inhibit the algae growth, and the cost of nutrients added and for biomass harvesting (Ahmad et al., 2021; Watanabe and Isdepsky, 2021).

## 3 Genetic engineering as promising tool to enhance bioremediation efficiencies

A possible option to improve the application of algae in industrial processes, such as PW treatment, could be strain improvement through genetic engineering (Ahmad et al., 2022). Even though advanced tools for genetic engineering have emerged at a great pace, they remain underutilized for microalgae as compared to other microorganisms. This is despite the demonstrated benefits of improving yields and overcoming challenges of high production costs (Bajhaiya et al., 2017; Kumar et al., 2020). In this section, we go over the different types of genetic modification which can be applied to algae, as well as how they can be used to improve PW bioremediation efforts.

### 3.1 Random mutagenesis

One of the powerful trait alteration tools for microalgae is random mutagenesis (Manandhar-Shrestha and Hildebrand, 2013; Arora et al., 2020). Obtaining mutants through random mutagenesis is accomplished through various methods, ranging from chemical, nuclear irradiation, plasmas, and ultraviolet (UV) mutagenesis. This, combined with a strong selective selection pressure, has proven to be a reliable strategy for producing strains with improved stress tolerances, resistance to contaminants, increased productivities (Cabanelas et al., 2016), but also higher metabolite production rates (Lai et al., 2004; Cordero et al., 2011; Doan and Obbard, 2012). Recently (Qi et al., 2018), developed a mutant of *Scenedesmus obliquus* for the purpose of increasing the CO_2_ bio-fixation and enhancing biomass production under elevated CO_2_ levels. The mutants with genetic stability as well as potential for increased CO_2_ tolerance were found through UV mutagenesis, as well as applying a low pH as a selective pressure. The authors found that the mutant strain was able to achieve higher biomass productivities and light conversion efficiencies under elevated CO_2_ concentrations compared to the parent strains, as well as contain 37% and 25% higher carbohydrate and lipid contents, respectively. Applying radon mutagenesis to algae strains such as those investigated by (Ammar et al., 2018), followed by exposure to high PW loadings, could potentially result in strains’ abilities to bioremediate PW without dilution, increasing its industrial and economic feasibility as a treatment option. Nevertheless, although interesting, the technology also exhibits some weaknesses, such as the fact that mutants may lose the mutation of interest within a number of generations, thus losing their enhanced trait, as well as the fact that experiments can have a long time span (Arora and Philippidis, 2021), making their application cumbersome.

### 3.2 Targeted genetic engineering

Besides random mutagenesis, using targeted genetic modification methods for heterologous expression of foreign genes is very promising for enhancing specific algae traits. Recently, genetic engineering has gained momentum because new and strong genetic tools are progressively offered and redesigning and improving metabolic pathways reveal new opportunities for the industrial development of microalgae (Ng et al., 2017). In the past decade, gene-editing tools such as (TALEN), (ZFN) (CRISPR/Cas9) technologies have emerged as the most popular recombinant DNA technologies that have been applied on a variety of different microorganisms, including microalgae (Fajardo et al., 2020; Kumar et al., 2020).

#### 3.2.1 Zinc-finger nucleases (ZFN)

**FIGURE 1:**
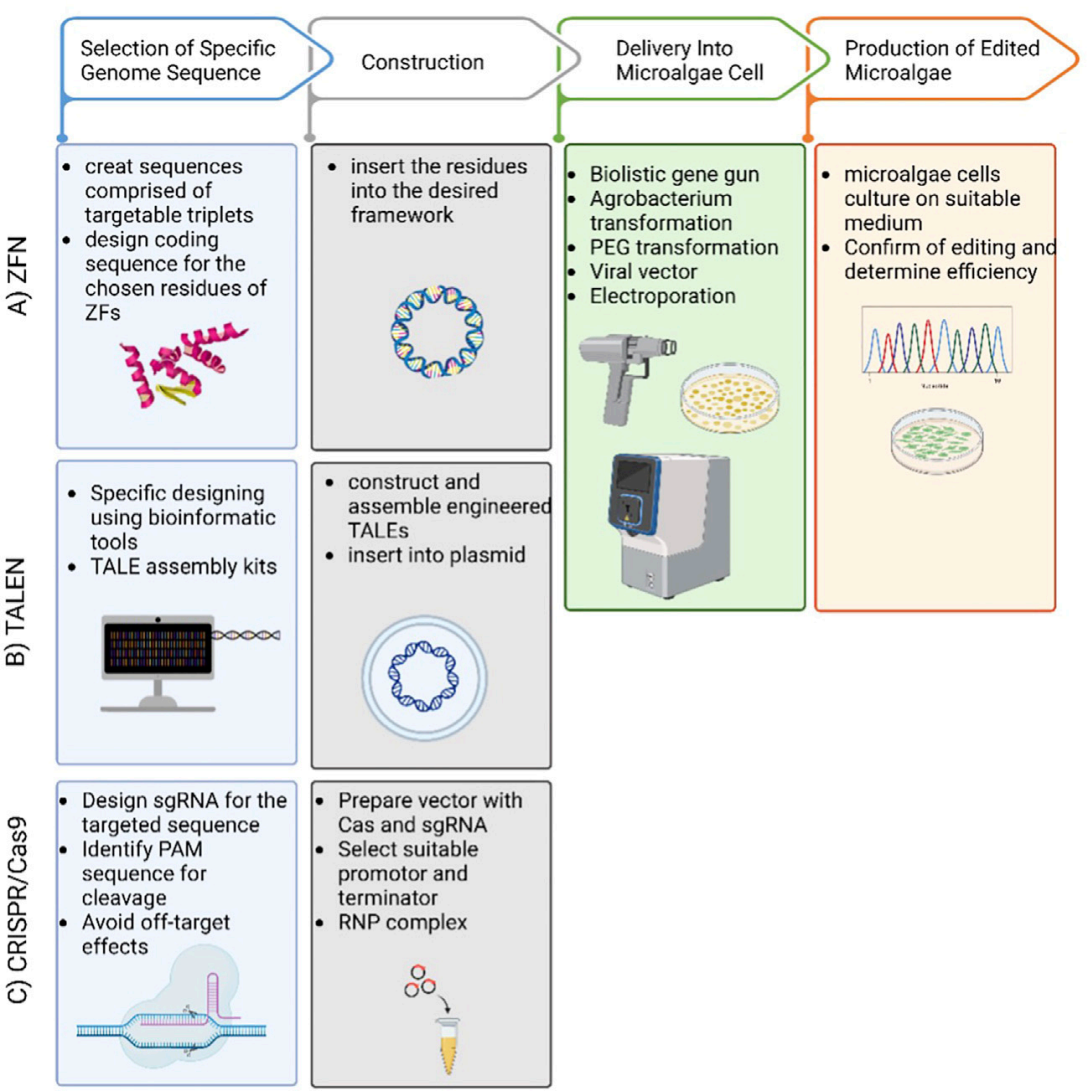
Simplified workflow for ZFN, TALEN and CRISPR/Cas9 mediated microalgae genome editing. **(A)** ZFN, **(B)** TALEN and **(C)** CRISPR/Cas9. The 3 tools have 4 steps (1) using bioinformatic tools to identify the targeted sequence and design accordingly, (2) construct the delivery method by choosing the vector, (3) select the delivery method to insert the plasmid into the microalgae cell, (4) subculture the microalgae on a selective medium and determine the efficiency of gene editing.

**FIGURE 2:**
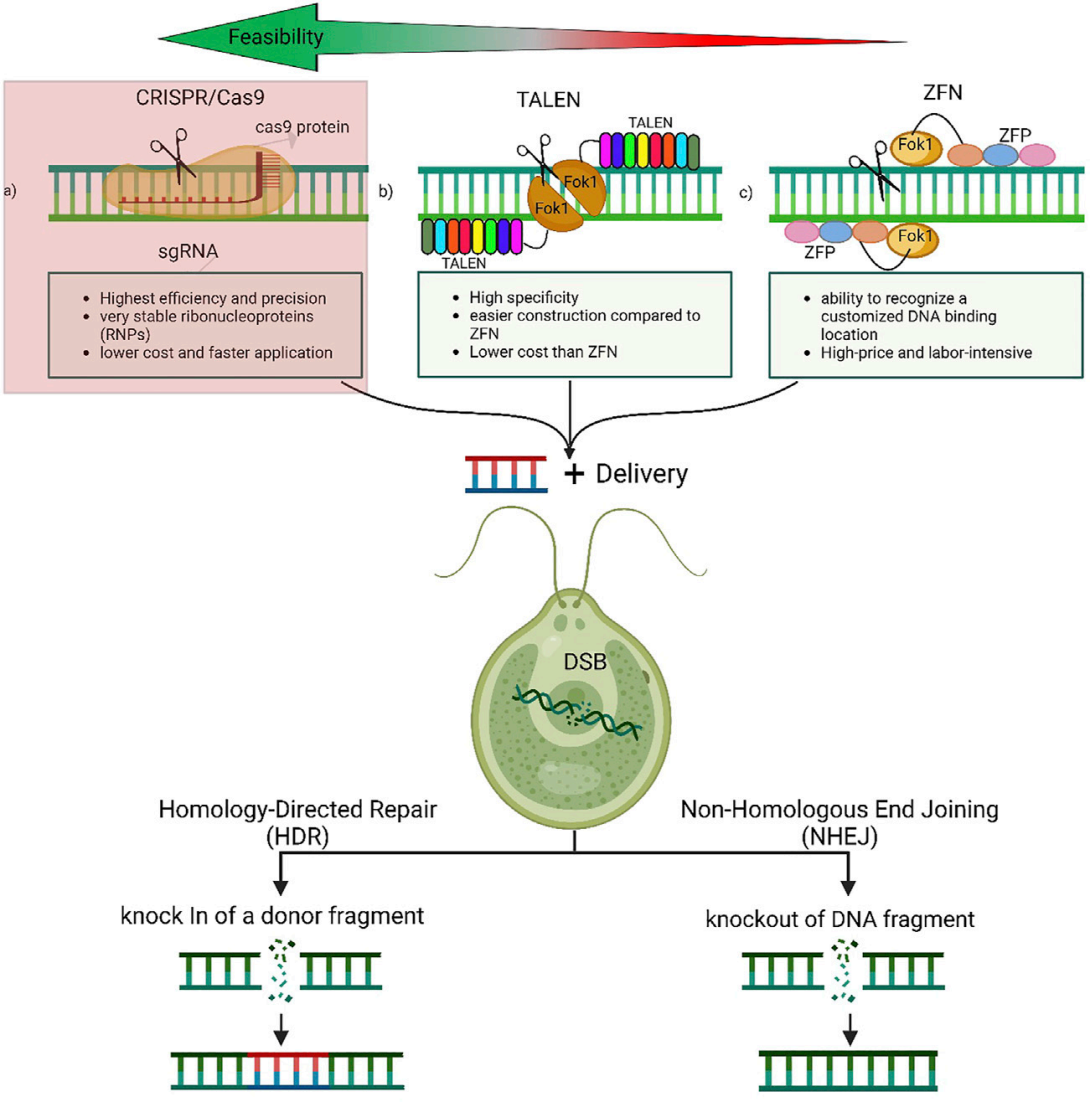
Genetic engineering tools-induced genome editing in Microalgae. The double-stranded breaks (DSBs) introduced at the target site by CRISPR/Cas or TALEN or ZFN complexes stimulates the endogenous DNA repair machineries, non-homologous end joining (NHEJ) in the absence of the donor template or the homology-directed repair (HDR) in presence of the donor template. The NHEJ is generally associated with the introduction of insertions and/or deletions (indels) of varying lengths at the DSB site, often leading to the disruption of the reading frame of the target gene. The HDR pathway results in a precise insertion or deletion at the DSB site by homologous recombination. The preferred tool is the CRISPR/Cas9 which is highlighted in red as it is very accurate, easy, and fast.

(Nain et al., 2010) describe zinc-finger nucleases (ZFNs) as a powerful tool that reshapes the boundaries of biological research. It is composed of programmable modules that bind to a specific DNA sequence. Excitingly, ZFN enables a wide range of genetic modifications by allowing DNA double-strand breaks that stimulate error repairs at specific genomic sites (Jabalameli et al., 2015; Kanchiswamy et al., 2015). The specificity of ZFNs arises from their versatility and ability to recognize a customized DNA binding location (Gaj et al., 2013) (Figure 2C). ZFNs have been used multiple times for chosen modifications of microalgal genomic DNA. It works by creating a cleavage site where insertions or deletions (INDELs) can take place (Jeon et al., 2017). ZFN technology was successfully applied to the model microalga *Chlamydomonas reinhardtii* in 2013 (Sizova et al., 2013). The ZFNs were created to target the *COP3* gene using paromomycin-resistance as a marker activity. Furthermore, this work proves that transient ZFN expression is not toxic for the cells as it results in stable transformed colonies. Similarly (Greiner et al., 2017), used ZFNs to reliably edit genes by homologous recombination in multiple strains of *Chlamydomonas*, including the wild type. This work also reported that promising results were achieved when the ZFN protocol was changed to replace glass beads with electroporation. Regardless of the numerous benefits of editing DNA with ZFNs, some issues may arise when it is applied. For example, there are limited sites to be targeted for nuclease selection. Also, there is a potential that double strand breaks may occur at an off-target site (Gupta and Musunuru, 2014). A simplified workflow of ZFN process is illustrated in (Figure 1).

#### 3.2.2 Transcription activator-like effector nucleases (TALEN)

Transcription activator-like effector nucleases (TALEN) are one of the first developed molecular editing tools (Zhang et al., 2016). TALEs are proteins that exist in nature in *Xanthomonas* bacteria (Malzahn et al., 2017), and are distinguished by their ability to recognize and bind to single base pairs of a DNA sequence (Gaj et al., 2013). When TALE proteins fuse with FokI nuclease, a double strand break is induced in the DNA, which enables knocking out genes or introducing mutations (Jeon et al., 2017) (Figure 1B). TALEN has many advantages, most importantly: i) it can be engineered to target a specific site in the genome; ii) it is much easier to design compared to ZFNs; iii) it is available commercially, as well as a TALEN-based library has been constructed; iv) it is not limited by the length of sequence it can bind to; v) fewer obstacles appear when selecting the binding site (Gupta and Musunuru, 2014). On the other hand, some constraints must be taken into consideration when applying TALEN. As such, compared to ZFNs, it appears to be much larger in size, knowing that the large size makes it less specific. Also, the larger the TALENs, the harder it becomes to deliver them to cells. Moreover, the host plasmid vector of the TALEN sequence tends to rearrange after transduction (Kumar et al., 2020). Several studies have reported the modification of the genomes of different microalgae. TALENs were used to enhance lipid metabolic pathways in the genome of the diatom *Phaeodactylum tricornutum*, according to (Daboussi et al., 2014) and (Hao et al., 2018). Likewise (Takahashi et al., 2018), enhanced the lipid content in the green microalga *Coccomyxa sp.* Another example of successful utilization of the platinum TALENs is efficiently mutating the nitrate reductase and acyltransferase genes in *Nannochloropsis oceanica* (Kurita et al., 2020). The process of applying TALEN in microalgae is shown in (Figure 1).

#### 3.2.3 CRISPR/Cas9

Due to its simplicity and versatility, CRISPR/cas9-mediated genome-editing is one of the most promising novel techniques for gene editing and has been shown to be successful in a variety of living organisms (Bortesi and Fischer, 2015; Xu M. et al., 2020). It offers an excellent time and labor efficient system (Doudna and Charpentier, 2014; Zhang et al., 2020) and multiple mitigation strategies succeeded in significantly reducing off-target effects (Han et al., 2020). Moreover, implementing a plasmid-free CRISPR-Cas9 system has resulted in very stable ribonucleoproteins (RNPs) in the studied cells (Song et al., 2019). To use genetically manipulated algae on an industrial scale, transformed cells need to demonstrate a stable gene expression on long term. Therefore, large scale algal cultivation require fully integrated gene cassettes within the genome the transformed strain (Patel et al., 2019). Many cases of CRISPR/Cas9 genetically manipulated microalgae have reported stable mutants, which may alleviate the problem of unsettled mutations (Nymark et al., 2016; Slattery et al., 2018). ((Nymark et al., 2016; Slattery et al., 2018). The mechanism of designing the CRISPR/Cas9 system to manipulate microalgae genome is expressed in (Figure 1) while its structure is shown in (Figure 2A)

The first successful application of CRISPR/Cas9 in microalgae was demonstrated in 2014, by (Jiang et al., 2014) on *Chlamydomonas reinhardtii*, although improvements were needed to reduce the cytotoxic effect of Cas9 on the algae strain (Baek et al., 2016; Shin et al., 2016). Since then, *Chlamydomonas* transformation using CRISPR system was accomplished multiple times, e.g., for increasing lipids and pigments content (Song et al., 2022), increasing triacylglycerol productivity (Lee et al., 2022); lipid accumulation (Nguyen T. H. T. et al., 2020); and for understanding CO_2_ sequestration mechanism (Asadian et al., 2022).

Due to its role in biofuel production; several studies have been conducted on algal cells for increasing lipid content through CRISPR/Cas9 genetic modification (D’Alessandro and Antoniosi Filho, 2016; Shokravi et al., 2020). For instance, a recent study by (Lin and Ng, 2020) investigated the improvement of the lipid content of *Chlorella vulgaris*, through targeted editing of the omega-3 fatty acid desaturase gene (*fad3*). Results showed that the genetically modified strain was able to reach up to 46% higher lipid content and 20% higher biomass concentrations, as compared to the wild type of strain. Other studies suggested that knocking out genes involved in the process of fatty acid degradation could induce increased lipid yields in different microalgal species (Nguyen A. D. et al., 2020; Chang et al., 2020). Several other studies were also successful in implementing CRISPR/Cas9 to increase the production of different carotenoids in *Dunaliella salina* (Hu et al., 2021); and in *Chlamydomonas reinhardtii* (Baek et al., 2016), to improve the thermal tolerance of *Tetraselmis suecica* (Xu J. et al., 2020), and to investigate gene function in *Phaeodactylum tricornutum* (Hao et al., 2022; Llavero-Pasquina et al., 2022). Overall, CRISPR/Cas9 has been successfully applied on many other algae species to improve their biomass productivities, tolerances to abiotic stressors, or increase lipid content, or that of other biomolecules and value-added products (Kumar et al., 2020; Jeon et al., 2021; Wang et al., 2021).

In summary, microalgae are emerging and thriving as sustainable base for biotechnology in general and produced wastewater treatment. Yet, it is not currently viable on the industrial scale due to many obstacles. Therefore, it is considered practical to use genetic engineering to enable the use of microalgae on a bigger scale. Furthermore, genome editing techniques may be used to further our understanding of the mechanisms behind the genes that enable microalgae to survive in such harmful environments. ZFNs, TALENs, and CRISPR/Cas9 were successfully implemented in enhancing various applications of microalgae including production of pharmaceutical products lipid, carotenoids, and protein (Grama et al., 2022). Several reviews summarized the major differences between these technologies stating the advantages and disadvantages of each individually (Jeon et al., 2017; Liu et al., 2022). These new approaches are of great interest to optimize specific traits of microalgae to boost their effectiveness in phytoremediation.

### 3.3 Applying CRISPR/Cas9 for improving PW treatment: Target genes

Although the application of CRISPR/Cas9 to enhance many microalgal traits, productivities, and production of primary metabolites has been increasing, there are very few studies that report the use of CRISPR/Cas9 to enhance microalgal applications in wastewater treatment (Patel et al., 2019; Feng et al., 2020). In general, there are a plethora of candidate genes that can alter metabolic pathways in favor of bioremediation of wastewaters, with the potential to improve bioremediation and biomass production (Balzano et al., 2020). To enhance microalgal PW treatment through genetic engineering, two strategies can be applied: (a) improving strain tolerance and degradability of certain pollutants, such as hydrocarbons or heavy metals, or (b) expressing and producing of degradation aiding molecules, such as surfactants or antifouling ingredients (Feng et al., 2020). In both cases, the first step is the identification of target genes for transformation into selected microalgae strains.

#### 3.3.1 Tolerance and accumulation of heavy metals

Various wastewaters contain hazardous and toxic pollutants that pose a significant environmental risk, including heavy metals (Gray, 1998). Furthermore, the removal of heavy metals from wastewater is a serious issue and can be challenging in many cases (Kaur and Roy, 2021). However, numerous algal strains have been found to be capable of sequestering metals through the use of extracellular polysaccharides (EPS) and intracellular polyphosphates, which chelate metal ions (Opeolu et al., 2010). Additionally, the potential use of genetically engineered microalgae for metal bioremediation is paving the way for more evaluations and selections of novel genes that are involved in metal accumulation (Cheng et al., 2019).

The most common genetic manipulation techniques for algal-based metal recovery are overexpression of genes and introducing exogenous DNA by constructing transgenic algal strains (Cheng et al., 2019). Interestingly, it is worth mentioning that numerous authors have discussed and studied the introduction of foreign DNA fragments to different organisms for the purpose of increasing their heavy metal tolerance, such as plants and bacteria; only few have highlighted this genetic manipulation strategy for microalgae. One such example in bacteria comes from (Sriprang et al., 2003), who introduced genes for phytochelating synthase (PCSAt) in *Mesorhizobium huakuiiwhich*. PCSAt is a protease-like enzyme that catalyzes the synthesis of peptides, which in turn chelate metals (Rigouin et al., 2013). It was reported that the transformation resulted in a transgenic strain that accumulated Cd^+2^ 9–19-fold more than the strain that did not contain the PCSAt. In another study, ACC deaminase and iaaM genes were introduced into the *Petunia hybrida* Vilm plant *via* agrobacterium-mediated transformation, and the transgenic plants were continuously treated with Copper (II) sulfate CuSO^4^ and Cobalt (II) chloride CoCl^2^ to test for heavy metal tolerance (Zhang et al., 2008). The authors planted the transgenic Petunia in heavily heavy metal contaminated soil and found that the mutant plant had double the growth of the wild-type plant. Also, they grew bigger, healthier, and faster in the heavy metal contaminated soil, most likely related to the increased tolerance to cobalt that resulted from the co-expression of both introduced genes. Other genes that could potentially improve heavy metal tolerance and accumulation were identified by (Peng et al., 2020) in the plant *Kandelia obovate*. This study showed that *KoCBF1* and *KoCBF3* genes were highly expressed in the presence of lead (Pb (NO_3_)^2^), implying that they are involved in growth and heavy metal accumulation. One of the few examples found in literature demonstrated that wild-type *C. reinhardtii* could not survive in the presence of Cadmium (Cd) in the cultivation media, whereas a transgenic strain with high expression level of the *CrMTP4* gene was able to grow well under the metal stress (Ibuot et al., 2017).

#### 3.3.2 Hydrocarbon degradation

It is well known that the composition of wastewater, including PW, varies greatly based on its origin, also applying to the level of TOC, including hydrocarbons. As an example, TOC in PW collected from a petroleum industrial site in Qatar was found to be 720.33 mg L^−1^ (Das et al., 2019), however, values of up to 2430 mg L^−1^ have also been reported (Shaikh et al., 2020).

Microbial degradation of hydrocarbons is a common phenomenon, although its applications in bioremediation are still limited (Ławniczak et al., 2020). These microorganisms can however be the source of relevant genes that are in control of hydrocarbon degradation and organic carbon digestion which could be applied in microalgae. For example (Luo et al., 2015), constructed the pCom8 vector to express alkane hydroxylase in *Escherichia. coli* (*E. coli*) DH5α, after which it was inoculated in diesel containing media to induce gene expression and perform biodegradation assays. Furthermore, applying a consortium of *Acinetobacter* and the transgenic *E. coli* strain improved diesel biodegradation by up to 49% compared to the control. Another study conducted by (Kang et al., 2017) cloned a two-component flavin-diffusible monooxygenase gene (*cph*) from *Arthrobacter chlorophenolicus* for enzyme overexpression. This enzyme is responsible for the degradation and removal of 4-chlorophenol (4-CP) and transformed strains could remove up to 82.7% of 4-CP from the media.

Besides the introduction of genes involved in hydrocarbon degradation, increasing biosurfactant production to aid degradation can be of great interest (Ochsner et al., 1994). Several microalgae and cyanobacteria were tested for their capability of producing biosurfactant. *Dunaliella salina* and *Porphyridium cruentum*, two marine microalgal species that produce extracellular polymeric substances that can be used as emulsifiers to metabolize oil hydrocarbons (Sukla et al., 2019). Also (Radmann et al., 2015), stated that *Arthrospira sp.* and other cyanobacteria and microalgae can produce biosurfactant as a by-product in the presence of certain organic carbon. In this context, genetic engineering can be helpful to increase the potential of microalgae to secrete biosurfactant. Numerous research efforts were made to understand their production on the molecular level, and the different genes involved in enhancing it were discovered. For instance, the *Emt1*, *Mmc1*, *Mac1*, *Mac2* genes in *Ustilago maydis* were studied for their association with the expression of Mannosylerythritol Lipids (MELs), which are class of biosurfactant (Markande et al., 2021). These lipids are also expressed in different microalgae (Luca et al., 2021). Moreover, using wastewater as a substrate for *Pseudozyma tsukubaensis* made it possible to successfully increase the production of MELs (Andrade et al., 2017).An overview of these and other reported genes that influence heavy metal and hydrocarbon degradation is given in Table 2. Using gene-editing technologies like CRISPR to apply such genes to microalgae could potentially increase the feasibility of using it for produced water treatment.

**TABLE 2:** Candidate genes for heavy metal tolerance and accumulation and hydrocarbon degradation.

Gene	Plasmid	Promotor	Application	Reference
*PCS*_*At*_	pBBR1MCS-2 pMP220	*nifH*	Accumulation of Cd^+2^	Sriprang et al. (2003)
*KoCBF3*			Accumulation of Pb	Peng et al. (2020)
*ACC* deaminase	pBI-iaaM/ACC	*CaMV 35S*	Accumulation of copper and cobalt	Zhang et al. (2008)
*iaaM*	pBI-iaaM	*GRP*	Accumulation of copper and cobalt	Zhang et al. (2008)
*CrMTP4*	CrMTP4gDNA-pH2GW7	Not mentioned	Increase Mn and Cd content in the cell	Ibuot et al. (2017)
alkane hydroxylase (*alkB*)	pCom8	Not mentioned	Degrading diesel oil	Luo et al. (2015)
*cph*	pET-24a	Not mentioned	Removal of 4-chlorophenol	Kang et al. (2017)
*mat1*	pET15b	—	Biosurfactant	Hewald et al. (2006)

In summation, microalgae can ideally contribute to the bioremediation of heavily contaminated water with heavy metals or organic pollutants. Owing to its fast adaptability to use these contaminants to thrive. Additionally, the role of genetic engineering to develop and understand the mechanism in which microalgae consume such pollutants cannot be ignored. Thus, to overcome the growing environmental threat of produced water on the sustainable development; an increased focused research is acquired to strengthen the large-scale use of genetically engineered microalgae and to fill the knowledge gap in this field.

### 3.4 Challenges and limitations of genetic engineering for enhancing bioremediation efficiencies

Many water treatment methods depend on using microbes that degrade pollutants. Recently, there has been a great focus on genetically modifying such microorganisms to augment their ability for bioremediation. Nevertheless, there are limitations and challenges linked to redesign microorganisms’ DNA related to gene expression efficiency and the stability of introduced genes according to (Fajardo et al., 2020) and (Tran and Kaldenhoff, 2020); the success of genetic transformation is highly dependent on the species selection. Some diatoms and microalgal species are known for their low stability after nuclear transformation such as *Thalassiosira weissflogii*, *Ulva lactuca*, and *Gracilaria changii* (Fajardo et al., 2020). Another major concern is the presence of off-target effects of CRISPR/Cas9(Zhang et al., 2015). Additionally, genome-editing techniques can be very expensive and involve complex procedures with some technical challenges, such as TALEN (Khan, 2019). Furthermore, in order to manipulate the genome of microalgae to improve its removal of a specific contaminant, such as heavy metals, a complete understanding of the cells' metabolism and structure during metal stress is required to ensure the maximum effectiveness of those engineered cells for bioremediation (Ranjbar and Malcata, 2022).

Other limitations of using genetically enhanced microalgae on a large-scale, include environmental concerns and issues related to public health (Janssen and Stucki, 2020; Kumar et al., 2020). Thus, the environmental impact assessments and other assessments that comprise biosafety must be performed prior to using a genetically modified organism (GMO) (Sayler and Ripp, 2000). Nevertheless, several successful studies were conducted about releasing transgenic organisms into the environment. For instance, the field trials of genetically engineered mosquitos in North America (Neuhaus, 2018), and the release of *Pseudomonas fluorescens HK44* in a controlled field in the US (Ripp et al., 2000). Also (De Leij et al., 1995), spread transgenic *Pseudomonas fluorescens* in a wheat field in 1995. Using GMOs in bioremediation poses a risk of horizontal gene transfer as well as regulatory and ecological complications (Singh et al., 2011).

Thus, even though this technology has the potential for future use, its sustainability and large-scale use are still in question. Optimization for a more sustainable use on a large scale should be considered. Finally, microalgae have great potential for many biotechnological applications, but to date, the development and improvement of industrial production using CRISPR/Cas9 or other biotechnologies has yet to be conducted and tested for feasible and satisfying outcomes.

## 4 Conclusion and recommendations

This review discussed the potential of using the genetic engineering tools ZFNs, TALENs, and CRISPR/Cas9 to manipulate the genome of microalgae. It is believed that they have a great opportunity to improve the tolerance of microalgae to toxic mediums like the produced water. As well as to enhance its ability to remove existing contaminants. Deeper investigation of these technologies is crucial the potential genes and understand their mechanism and function for bioremediation. However, CRISPR/Cas9, the latest and most promising tool for genome modification is believed to hold applicational advantages over the other technologies as it offers more stability to the introduced gene; and it consumes less time and effort with fewer technical issues.

Therefore, it is highly recommended to extend the research on CRISPR/Cas9 application to serve the purpose of bioremediation of produced water using microalgae given that they are efficient and economically feasible candidate to make the PW reusable and lower its negative impact on the environment. Finally, more research effort is required to overcome the problems that arise when CRISPR/Cas9 is being applied such as the off-target effects.

## Abbreviations

CCU, carbon capture and utilization; Cd, Cadmium; CO_2,_ carbon dioxide; CoCl^2^, Cobalt (II) chloride; COD, chemical oxygen demand; CRISPR, clustered regularly interspaced short palindromic repeats; CuSO^4^, Copper (II) sulfate; EPS, extracellular polysaccharides; GMO, genetically modified organism; INDELs, insertions or deletions; NORM, naturally occurring radioactive material; PW, Produced water; RNPs, ribonucleoproteins; TALEN, Transcription activator-like effector nuclease; TDS, total dissolved solids; TN, Total Nitrogen; TOC, Total Organic Carbon; UV, ultraviolet; ZFN, Zinc finger nuclease.

## References

[B1] Ahmad A. Banat F. Alsafar H. Hasan S. W. (2022). Algae biotechnology for industrial wastewater treatment, bioenergy production, and high-value bioproducts. Sci. Total Environ. 806, 150585. 10.1016/j.scitotenv.2021.150585 34597562

[B2] Ahmad I. Abdullah N. Koji I. Yuzir A. Mohamad S. E. (2021). Potential of microalgae in bioremediation of wastewater. Bull. Chem. React. Eng. 16, 413–429. 10.9767/bcrec.16.2.10616.413-429

[B3] Ahmad I. Abdullah N. Koji I. Yuzir A. Mohamad S. E. (2021). Potential of microalgae in bioremediation of wastewater. Bull. Chem. React. Eng. Catal. 16, 413–429. 10.9767/bcrec.16.2.10616.413-429

[B4] Al-Ghouti M. A. Al-Kaabi M. A. Ashfaq M. Y. Da’na D. A. (2019). Produced water characteristics, treatment and reuse: A review. J. Water Process Eng. 28, 222–239. 10.1016/j.jwpe.2019.02.001

[B6] Al-Jabri H. Das P. Khan S. Thaher M. Abdulquadir M. (2021). Treatment of wastewaters by microalgae and the potential applications of the produced biomass—A review. WaterSwitzerl. 13, 27. 10.3390/w13010027

[B7] Alalawy A. I. A. Sh Alabdraba W. M. Omer E. A. (2019). Nutrients and organic matters removal of ospitals wastewater by microalgae. J. Phys. Conf. Ser. 1294, 072002. 10.1088/1742-6596/1294/7/072002

[B8] Alsarayreh M. Almomani F. Khraisheh M. Nasser M. S. Soliman Y. (2022). Biological-based produced water treatment using microalgae: Challenges and efficiency. Sustainability 14 (1), 499. 10.3390/su14010499

[B9] Ammar S. H. Khadim H. J. Isam A. (2018). Cultivation of Nannochloropsis oculata and Isochrysis galbana microalgae in produced water for bioremediation and biomass production. Environ. Technol. Innovation 10, 132–142. 10.1016/j.eti.2018.02.002

[B10] Andrade C. J. de B, L. M. de A. Rocco S. A. Sforça M. L. Pastore G. M. Jauregi P. (2017). A novel approach for the production and purification of mannosylerythritol lipids (MEL) by Pseudozyma tsukubaensis using cassava wastewater as substrate. Sep. Purif. Technol. 180, 157–167. 10.1016/j.seppur.2017.02.045

[B11] Arias D. M. Uggetti E. García J. (2020). Assessing the potential of soil cyanobacteria for simultaneous wastewater treatment and carbohydrate-enriched biomass production. Algal Res. 51, 102042. 10.1016/j.algal.2020.102042

[B12] Arora N. Philippidis G. P. (2021). Microalgae strain improvement strategies: Random mutagenesis and adaptive laboratory evolution. Trends Plant Sci. 26, 1199–1200. 10.1016/j.tplants.2021.06.005 34226108

[B13] Arora N. Yen H.-W. Philippidis G. P. (2020). Harnessing the power of mutagenesis and adaptive laboratory evolution for high lipid production by oleaginous microalgae and yeasts. Sustainability 12, 5125. 10.3390/su12125125

[B14] Asadian M. Saadati M. Bajestani F. B. Beardall J. Abdolahadi F. Mahdinezhad N. (2022). Knockout of Cia5 gene using CRISPR/Cas9 technique in Chlamydomonas reinhardtii and evaluating CO2 sequestration in control and mutant isolates. J. Genet. 101, 6. 10.1007/s12041-021-01350-x 35129125

[B15] Baek K. Kim D. H. Jeong J. Sim S. J. Melis A. Kim J.-S. (2016). DNA-free two-gene knockout in Chlamydomonas reinhardtii via CRISPR-Cas9 ribonucleoproteins. Sci. Rep. 6, 30620. 10.1038/srep30620 27466170 PMC4964356

[B16] Bajhaiya A. K. Ziehe Moreira J. Pittman J. K. (2017). Transcriptional engineering of microalgae: Prospects for high-value chemicals. Trends Biotechnol. 35, 95–99. 10.1016/j.tibtech.2016.06.001 27387061

[B17] Balzano S. Sardo A. Blasio M. Chahine T. B. Dell’Anno F. Sansone C. (2020). Microalgal metallothioneins and phytochelatins and their potential use in bioremediation. Front. Microbiol. 11, 517. 10.3389/fmicb.2020.00517 32431671 PMC7216689

[B18] Bortesi L. Fischer R. (2015). The CRISPR/Cas9 system for plant genome editing and beyond. Biotechnol. Adv. 33, 41–52. 10.1016/j.biotechadv.2014.12.006 25536441

[B19] Cabanelas I. T. D. van der Zwart M. Kleinegris D. M. M. Wijffels R. H. Barbosa M. J. (2016). Sorting cells of the microalga Chlorococcum littorale with increased triacylglycerol productivity. Biotechnol. Biofuels 9, 183. 10.1186/s13068-016-0595-x 27582875 PMC5006580

[B20] Calderón-delgado I. C. Mora-solarte D. A. Velasco-Santamaría Y. M. (2019). Physiological and enzymatic responses of Chlorella vulgaris exposed to produced water and its potential for bioremediation. Environ. Monit. Assess. 191, 399. 10.1007/s10661-019-7519-8 31134347

[B21] Chang K. S. Kim J. Park H. Hong S. J. Lee C. G. Jin E. S. (2020). Enhanced lipid productivity in AGP knockout marine microalga Tetraselmis sp. using a DNA-free CRISPR-Cas9 RNP method. Bioresour. Technol. 303, 122932. 10.1016/j.biortech.2020.122932 32058903

[B22] Cheng S. Y. Show P. Lau F. Chang J. Ling T. C. (2019). New prospects for modified algae in heavy metal adsorption. Trends Biotechnol. 37, 1255–1268. 10.1016/j.tibtech.2019.04.007 31174882

[B23] Cho S. Luong T. T. Lee D. Oh Y. K. Lee T. (2011). Reuse of effluent water from a municipal wastewater treatment plant in microalgae cultivation for biofuel production. Bioresour. Technol. 102, 8639–8645. 10.1016/j.biortech.2011.03.037 21474308

[B24] Cordero B. F. Obraztsova I. Couso I. Leon R. Vargas M. A. Rodriguez H. (2011). Enhancement of lutein production in Chlorella sorokiniana (chorophyta) by improvement of culture conditions and random mutagenesis. Mar. Drugs 9, 1607–1624. 10.3390/md9091607 22131961 PMC3225938

[B25] Daboussi F. Leduc S. Maréchal A. Dubois G. Guyot V. Perez-Michaut C. (2014). Genome engineering empowers the diatom Phaeodactylum tricornutum for biotechnology. Nat. Commun. 5, 3831–3837. 10.1038/ncomms4831 24871200

[B26] D’Alessandro E. B. Antoniosi Filho N. R. (2016). Concepts and studies on lipid and pigments of microalgae: A review. Renew. Sustain. Energy Rev. 58, 832–841. 10.1016/j.rser.2015.12.162

[B27] Das P. AbdulQuadir M. Thaher M. Khan S. Chaudhary A. K. Alghasal G. (2019). Microalgal bioremediation of petroleum-derived low salinity and low pH produced water. J. Appl. Phycol. 31, 435–444. 10.1007/s10811-018-1571-6

[B28] De Leij F. A. A. M. Sutton E. J. Whipps J. M. Fenlon J. S. Lynch J. M. (1995). Field release of a genetically modified Pseudomonas fluorescens on wheat: Establishment, survival and dissemination. Bio/Technology 13, 1488–1492. 10.1038/nbt1295-1488

[B29] Devi N. D. Sun X. Ding L. Goud V. V. Hu B. (2022). Mixotrophic growth regime of novel strain Scenedesmus sp. DDVG I in municipal wastewater for concomitant bioremediation and valorization of biomass. J. Clean. Prod. 365, 132834. 10.1016/j.jclepro.2022.132834

[B30] Deviram G. Mathimani T. Anto S. Shan T. Arul D. Pugazhendhi A. (2020). Applications of microalgal and cyanobacterial biomass on a way to safe, cleaner and a sustainable environment. J. Clean. Prod. 253, 119770. 10.1016/j.jclepro.2019.119770

[B31] Doan T. T. Y. Obbard J. P. (2012). Enhanced intracellular lipid in Nannochloropsis sp. via random mutagenesis and flow cytometric cell sorting. Algal Res. 1, 17–21. 10.1016/j.algal.2012.03.001

[B32] Doudna J. A. Charpentier E. (2014). Genome editing. The new frontier of genome engineering with CRISPR-Cas9. Science 346, 1258096. 10.1126/science.1258096 25430774

[B33] El-Kassas H. Y. Mohamed L. A. (2014). Bioremediation of the textile waste effluent by Chlorella vulgaris. Egypt. J. Aquatic Res. 40, 301–308. 10.1016/j.ejar.2014.08.003

[B34] Fajardo C. De Donato M. Carrasco R. Martínez-Rodríguez G. Mancera J. M. Fernández-Acero F. J. (2020). Advances and challenges in genetic engineering of microalgae. Rev. Aquac. 12, 365–381. 10.1111/raq.12322

[B35] Feng S. Hu L. Zhang Q. Zhang F. Du J. Liang G. (2020). CRISPR/Cas technology promotes the various application of Dunaliella salina system. Appl. Microbiol. Biotechnol. 104, 8621–8630. 10.1007/s00253-020-10892-6 32918585

[B36] Gaj T. Gersbach C. A. Barbas C. F. (2013). ZFN, TALEN, and CRISPR/Cas-based methods for genome engineering. Trends Biotechnol. 31, 397–405. 10.1016/j.tibtech.2013.04.004 23664777 PMC3694601

[B37] Gimpel J. A. Henríquez V. Mayfield S. P. (2015). In metabolic engineering of eukaryotic microalgae: Potential and challenges come with great diversity. Front. Microbiol. 6, 1376. 10.3389/fmicb.2015.01376 26696985 PMC4678203

[B38] Grama S. B. Liu Z. Li J. (2022). Emerging trends in genetic engineering of microalgae for commercial applications. Mar. Drugs 20, 285. 10.3390/md20050285 35621936 PMC9143385

[B39] Gray M. (2020). “Reuse of produced water in the oil and gas industry,” in SPE International Conference and Exhibition on Health, Safety, Environment and Sustainability SPE-199498-MSy, England, July 20 2020.

[B40] Gray S. N. (1998). Fungi as potential bioremediation agents in soil contaminated with heavy or radioactive metals. Biochem. Soc. Trans. 26, 666–670. 10.1042/bst0260666 10047803

[B41] Greiner A. Kelterborn S. Evers H. Kreimer G. Sizova I. Hegemann P. (2017). Targeting of photoreceptor genes in Chlamydomonas reinhardtii via zinc-finger nucleases and CRISPR/Cas9. Plant Cell 29, 2498–2518. 10.1105/tpc.17.00659 28978758 PMC5774583

[B42] Gupta R. M. Musunuru K. (2014). Expanding the genetic editing tool kit: ZFNs, TALENs, and CRISPR-cas9. J. Clin. Investigation 124, 4154–4161. 10.1172/JCI72992 PMC4191047 25271723

[B43] Han H. A. Pang J. K. S. Soh B. S. (2020). Mitigating off-target effects in CRISPR/Cas9-mediated *in vivo* gene editing. J. Mol. Med. 98, 615–632. 10.1007/s00109-020-01893-z 32198625 PMC7220873

[B44] Hao X. Chen W. Amato A. Jouhet J. Maréchal E. Moog D. (2022). Multiplexed CRISPR/Cas9 editing of the long‐chain acyl‐CoA synthetase family in the diatom *Phaeodactylum tricornutum* reveals that mitochondrial ptACSL3 is involved in the synthesis of storage lipids. New Phytol. 233, 1797–1812. 10.1111/nph.17911 34882804

[B45] Hao X. Luo L. Jouhet J. Rébeillé F. Maréchal E. Hu H. (2018). Enhanced triacylglycerol production in the diatom Phaeodactylum tricornutum by inactivation of a Hotdog-fold thioesterase gene using TALEN-based targeted mutagenesis. Biotechnol. Biofuels 11, 312–319. 10.1186/s13068-018-1309-3 30455741 PMC6231261

[B46] Hewald S. Linne U. Scherer M. Marahiel M. A. Bo M. Bölker M. (2006). Identification of a gene cluster for biosynthesis of mannosylerythritol lipids in the basidiomycetous fungus Ustilago maydis. Appl. Environ. Microbiol. 72, 5469–5477. 10.1128/AEM.00506-06 16885300 PMC1538720

[B47] Hopkins T. C. Graham E. J. S. Schuler A. J. (2019). Biomass and lipid productivity of Dunaliella tertiolecta in a produced water-based medium over a range of salinities. J. Appl. Phycol. 31, 3349–3358. 10.1007/s10811-019-01836-3

[B48] Hu L. Feng S. Liang G. Du J. Li A. Niu C. (2021). CRISPR/Cas9-induced β-carotene hydroxylase mutation in Dunaliella salina CCAP19/18. Amb. Express 11, 83. 10.1186/s13568-021-01242-4 34097133 PMC8185118

[B49] Huang K. Chen C. Shen Q. Rosen B. P. Zhao F.-J. (2015). Genetically engineering Bacillus subtilis with a heat-resistant arsenite methyltransferase for bioremediation of arsenic-contaminated organic waste. Appl. Environ. Microbiol. 81, 6718–6724. 10.1128/AEM.01535-15 26187966 PMC4561676

[B50] Ibuot A. Dean A. P. McIntosh O. A. Pittman J. K. (2017). Metal bioremediation by CrMTP4 over-expressing Chlamydomonas reinhardtii in comparison to natural wastewater-tolerant microalgae strains. Algal Res. 24, 89–96. 10.1016/j.algal.2017.03.002

[B51] Jabalameli H. R. Zahednasab H. Karimi-Moghaddam A. Jabalameli M. R. (2015). Zinc finger nuclease technology: Advances and obstacles in modelling and treating genetic disorders. Gene 558, 1–5. 10.1016/j.gene.2014.12.044 25536166

[B52] Janssen D. B. Stucki G. (2020). Perspectives of genetically engineered microbes for groundwater bioremediation. Environ. Sci. Process. Impacts 22, 487–499. 10.1039/c9em00601j 32095798

[B53] Japar A. S. Takriff M. S. Mohd Yasin N. H. (2021). Microalgae acclimatization in industrial wastewater and its effect on growth and primary metabolite composition. Algal Res. 53, 102163. 10.1016/j.algal.2020.102163

[B54] Jayakumar S. Bhuyar P. Pugazhendhi A. Rahim M. H. A. Maniam G. P. Govindan N. (2021). Effects of light intensity and nutrients on the lipid content of marine microalga (diatom) Amphiprora sp. for promising biodiesel production. Sci. Total Environ. 768, 145471. 10.1016/j.scitotenv.2021.145471 33736330

[B55] Jeon M. S. Han S.-I. Jeon M. Choi Y.-E. (2021). Enhancement of phycoerythrin productivity in Porphyridium purpureum using the clustered regularly interspaced short palindromic repeats/CRISPR-associated protein 9 ribonucleoprotein system. Bioresour. Technol. 330, 124974. 10.1016/j.biortech.2021.124974 33743273

[B56] Jeon S. Lim J. M. Lee H. G. Shin S. E. Kang N. K. Park Y. Il (2017). Current status and perspectives of genome editing technology for microalgae. Biotechnol. Biofuels 10, 267. 10.1186/s13068-017-0957-z 29163669 PMC5686953

[B57] Jiang W. Brueggeman A. J. Horken K. M. Plucinak T. M. Weeks D. P. (2014). Successful transient expression of Cas9 and single guide RNA genes in Chlamydomonas reinhardtii. Eukaryot. Cell 13, 1465–1469. 10.1128/EC.00213-14 25239977 PMC4248704

[B58] Kalra R. Gaur S. Goel M. (2021). Microalgae bioremediation: A perspective towards wastewater treatment along with industrial carotenoids production. J. Water Process Eng. 40, 101794. 10.1016/j.jwpe.2020.101794

[B59] Kang C. Yang J. W. Cho W. Kwak S. Park S. Lim Y. (2017). Oxidative biodegradation of 4-chlorophenol by using recombinant monooxygenase cloned and overexpressed from Arthrobacter chlorophenolicus A6. Bioresour. Technol. 240, 123–129. 10.1016/j.biortech.2017.03.078 28343861

[B60] Kaur S. Roy A. (2021). Bioremediation of heavy metals from wastewater using nanomaterials. Environ. Dev. Sustain. 23, 9617–9640. 10.1007/s10668-020-01078-1

[B61] Khan S. H. (2019). Genome-editing technologies: Concept, pros, and cons of various genome-editing techniques and bioethical concerns for clinical application. Mol. Ther. - Nucleic Acids 16, 326–334. 10.1016/j.omtn.2019.02.027 30965277 PMC6454098

[B62] Kumar G. Shekh A. Jakhu S. Sharma Y. Kapoor R. (2020). Bioengineering of microalgae: Recent advances, perspectives, and regulatory challenges for industrial application. Front. Bioeng. Biotechnol. 8, 914. 10.3389/fbioe.2020.00914 33014997 PMC7494788

[B63] Kurita T. Moroi K. Iwai M. Okazaki K. Shimizu S. Nomura S. (2020). Efficient and multiplexable genome editing using Platinum TALENs in oleaginous microalga, Nannochloropsis oceanica NIES-2145. Genes Cells 25, 695–702. 10.1111/gtc.12805 32888368

[B64] Lai Y.-P. Huang J. Wang L.-F. Jun Li Z.-R. W. Wu Z. R. (2004). A new approach to random mutagenesis *in vitro* . Biotechnol. Bioeng. 86, 622–627. 10.1002/bit.20066 15137072

[B65] Ławniczak Ł. Woźniak‐Karczewska M. Loibner A. P. Heipieper H. J. Chrzanowski Ł. (2020). Microbial degradation of hydrocarbons—basic principles for bioremediation: A review. Molecules 25 (4), 856. 10.3390/molecules25040856 32075198 PMC7070569

[B66] Lee Y. Rudolph P. Miller S. M. Li Y. (2022). Genetic compensation of triacylglycerol biosynthesis in the green microalga *Chlamydomonas reinhardtii* . Plant J. 111, 1069–1080. 10.1111/tpj.15874 35727866 PMC9545326

[B67] Leite L. de S. Hoffmann M. T. Daniel L. A. (2019). Microalgae cultivation for municipal and piggery wastewater treatment in Brazil. J. Water Process Eng. 31, 100821–100827. 10.1016/j.jwpe.2019.100821

[B68] Leng L. Wei L. Xiong Q. Xu S. Li W. Lv S. (2020). Use of microalgae based technology for the removal of antibiotics from wastewater: A review. Chemosphere 238, 124680. 10.1016/j.chemosphere.2019.124680 31545213

[B69] Li M. Zamyadi A. Zhang W. Dumée L. F. Gao L. (2022). Algae-based water treatment: A promising and sustainable approach. J. Water Process Eng. 46, 102630. 10.1016/j.jwpe.2022.102630

[B70] Lin W. R. Ng I. S. (2020). Development of CRISPR/Cas9 system in Chlorella vulgaris FSP-E to enhance lipid accumulation. Enzyme Microb. Technol. 133, 109458. 10.1016/j.enzmictec.2019.109458 31874693

[B71] Liu K. Chen R. Yang R. Chen Y. Zhu C. Tang Y. (2022). “Genome editing approaches applied to microalgae-based fuels,” in 3rd generation biofuels (Netherlands: Elsevier), 47–64. 10.1016/B978-0-323-90971-6.00013-9

[B72] Llavero‐Pasquina M. Geisler K. Holzer A. Mehrshahi P. Mendoza‐Ochoa G. I. Newsad S. A. (2022). Thiamine metabolism genes in diatoms are not regulated by thiamine despite the presence of predicted riboswitches. New Phytol. 235, 1853–1867. 10.1111/nph.18296 35653609 PMC9544697

[B73] Luca M. D. Pappalardo I. Limongi A. R. Viviano E. Radice R. P. Todisco S. (2021). Lipids from microalgae for cosmetic applications. Cosmetics 8, 52. 10.3390/cosmetics8020052

[B74] Luo Q. He Y. Hou D. Y. Zhang J. G. Shen X. R. (2015). GPo1 <italic&gt;alkB&lt;/italic&gt; gene expression for improvement of the degradation of diesel oil by a bacterial consortium. Braz. J. Microbiol. 46, 649–657. 10.1590/S1517-838246320120226 26413044 PMC4568855

[B75] Malzahn A. Lowder L. Qi Y. (2017). Plant genome editing with TALEN and CRISPR. Cell & Biosci. 7, 21. 10.1186/s13578-017-0148-4 PMC5404292 28451378

[B76] Manandhar-Shrestha K. Hildebrand M. (2013). Development of flow cytometric procedures for the efficient isolation of improved lipid accumulation mutants in a Chlorella sp. microalga. J. Appl. Phycol. 25, 1643–1651. 10.1007/s10811-013-0021-8 24273383 PMC3825614

[B77] Markande A. R. Patel D. Varjani S. (2021). A review on biosurfactants: Properties, applications and current developments. Bioresour. Technol. 330, 124963. 10.1016/j.biortech.2021.124963 33744735

[B78] Marques I. M. Oliveira A. C. V. de Oliveira O. M. C. Sales E. A. Moreira Í. T. A. (2021). A photobioreactor using Nannochloropsis oculata marine microalgae for removal of polycyclic aromatic hydrocarbons and sorption of metals in produced water. Chemosphere 281, 130775. 10.1016/j.chemosphere.2021.130775 34015656

[B79] Mehariya S. Goswami R. K. Verma P. Lavecchia R. Zuorro A. (2021). Integrated approach for wastewater treatment and biofuel production in microalgae biorefineries. Energies 14, 2282. 10.3390/en14082282

[B80] Molazadeh M. Ahmadzadeh H. Pourianfar H. R. Lyon S. Rampelotto P. H. (2019). The use of microalgae for coupling wastewater treatment with CO2 biofixation. Front. Bioeng. Biotechnol. 7, 42. 10.3389/fbioe.2019.00042 30941348 PMC6433782

[B81] Nagamangala Kanchiswamy C. Sargent D. J. Velasco R. Maffei M. E. Malnoy M. (2015). Looking forward to genetically edited fruit crops. Trends Biotechnol. 33, 62–64. 10.1016/j.tibtech.2014.07.003 25129425

[B82] Nain V. Sahi S. Verma A. (2010). CPP-ZFN: A potential DNA-targeting anti-malarial drug. Malar. J. 9, 258. 10.1186/1475-2875-9-258 20846404 PMC2949742

[B83] Neuhaus C. P. (2018). Community engagement and field trials of genetically modified insects and animals. Hastings Cent. Rep. 48, 25–36. 10.1002/hast.808 29457234

[B84] Ng I. Tan S. Kao P. Chang Y. Chang J. (2017). Recent developments on genetic engineering of microalgae for biofuels and bio-based chemicals. biotechnology 12, 1600644. 10.1002/biot.201600644 28786539

[B85] Nguyen A. D. Kim D. Lee E. Y. (2020a). Unlocking the biosynthesis of sesquiterpenoids from methane via the methylerythritol phosphate pathway in methanotrophic bacteria, using α-humulene as a model compound. Metab. Eng. 61, 69–78. 10.1016/j.ymben.2020.04.011 32387228

[B86] Nguyen H. T. Yoon Y. Ngo H. H. Jang A. (2021). The application of microalgae in removing organic micropollutants in wastewater. Crit. Rev. Environ. Sci. Technol. 51, 1187–1220. 10.1080/10643389.2020.1753633

[B87] Nguyen T. H. T. Park S. Jeong J. Shin Y. S. Sim S. J. Jin E. (2020b). Enhancing lipid productivity by modulating lipid catabolism using the CRISPR-Cas9 system in Chlamydomonas. J. Appl. Phycol. 32, 2829–2840. 10.1007/s10811-020-02172-7

[B88] Nymark M. Sharma A. K. Sparstad T. Bones A. M. Winge P. (2016). A CRISPR/Cas9 system adapted for gene editing in marine algae. Sci. Rep. 6, 24951. 10.1038/srep24951 27108533 PMC4842962

[B89] Ochsner U. Koch A. Fiechter A. Reiser J. (1994). Isolation and characterization of a regulatory gene affecting rhamnolipid biosurfactant synthesis in *Pseudomonas aeruginosa* . J. Bacteriol. 176, 2044–2054. 10.1128/jb.176.7.2044-2054.1994 8144472 PMC205310

[B90] Opeolu B. O. Bamgbose O. Arowolo T. A. Adetunji M. T. (2010). Utilization of biomaterials as adsorbents for heavy metals removal from aqueous matrices. Sci. Res. Essays 5, 1780–1787. 10.5897/SRE.9000983

[B91] Parsy A. Sambusiti C. Baldoni-andrey P. Elan T. Périé F. (2020). Cultivation of Nannochloropsis oculata in saline oil & gas wastewater supplemented with anaerobic digestion effluent as nutrient source. Algal Res. 50, 101966. 10.1016/j.algal.2020.101966

[B92] Patel V. K. Soni N. Prasad V. Sapre A. Dasgupta S. Bhadra B. (2019). CRISPR–Cas9 system for genome engineering of photosynthetic microalgae. Mol. Biotechnol. 61, 541–561. 10.1007/s12033-019-00185-3 31140149

[B93] Pathak B. Gupta S. Verma R. (2018). “Biosorption and biodegradation of polycyclic aromatic hydrocarbons (PAHs) by microalgae,” in *Green Adsorbents for pollutant removal* environmental chemistry for a sustainable world. Editors Crini G. Lichtfouse E. (Cham: Springer International Publishing), 215–247. 10.1007/978-3-319-92111-2_7

[B94] Peng Y. L. Wang Y. S. Fei J. Sun C. C. (2020). Isolation and expression analysis of two novel C-repeat binding factor (CBF) genes involved in plant growth and abiotic stress response in mangrove Kandelia obovata. Ecotoxicol. Lond. Engl. 29, 718–725. 10.1007/s10646-020-02219-y 32394360

[B95] Qi F. Wu D. Mu R. Zhang S. Xu X. (2018). Characterization of a microalgal UV mutant for CO 2 biofixation and biomass production. BioMed Res. Int. 2018, 1–8. 10.1155/2018/4375170 PMC6323505 30671452

[B96] Radakovits R. Jinkerson R. E. Darzins A. Posewitz M. C. (2010). Genetic engineering of algae for enhanced biofuel production. Eukaryot. Cell 9, 486–501. 10.1128/EC.00364-09 20139239 PMC2863401

[B97] Radmann E. M. Morais E. G. De Oliveira C. F. Jorge A. V. C. Alberto J. Costa V. (2015). Microalgae cultivation for biosurfactant production. Afr. J. Microbiol. Res. 9, 2283–2289. 10.5897/AJMR2015.7634

[B98] Rahman A. Agrawal S. Nawaz T. Pan S. Selvaratnam T. (2020). A review of algae-based produced water treatment for biomass and biofuel production. WaterSwitzerl. 12 (9), 2351. 10.3390/W12092351

[B99] Rahman A. Pan S. Houston C. Selvaratnam T. (2021). Evaluation of galdieria sulphuraria and chlorella vulgaris for the bioremediation of produced water. WaterSwitzerl. 13 (9), 1183. 10.3390/w13091183

[B100] Rahmani A. Zerrouki D. Tabchouche A. Djafer L. (2022). Oilfield - produced water as a medium for the growth of Chlorella pyrenoidosa outdoor in an arid region. Environ. Sci. Pollut. Res. 29, 87509–87518. 10.1007/s11356-022-21916-1 35809171

[B101] Ranjbar S. Malcata F. X. (2022). Is genetic engineering a route to enhance microalgae-mediated bioremediation of heavy metal-containing effluents? Molecules 27, 1473. 10.3390/molecules27051473 35268582 PMC8911655

[B102] Rigouin C. Nylin E. Cogswell A. A. Schaumlöffel D. Dobritzsch D. Williams D. L. (2013). Towards an understanding of the function of the phytochelatin synthase of schistosoma mansoni. PLoS Neglected Trop. Dis. 7, e2037. 10.1371/journal.pntd.0002037 PMC3561135 23383357

[B103] Ripp S. Nivens D. E. Ahn Y. Werner C. Jarrell J. Easter J. P. (2000). Controlled field release of a bioluminescent genetically engineered microorganism for bioremediation process monitoring and control. Environ. Sci. Technol. 34, 846–853. 10.1021/es9908319

[B104] Saadaoui I. Sedky R. Rasheed R. Bounnit T. Almahmoud A. Elshekh A. (2019). Assessment of the algae-based biofertilizer influence on date palm (Phoenix dactylifera L.) cultivation. J. Appl. Phycol. 31, 457–463. 10.1007/s10811-018-1539-6

[B105] Salgueiro J. L. Pérez L. Maceiras R. Sánchez A. Cancela A. (2016). Bioremediation of wastewater using chlorella vulgaris microalgae: Phosphorus and organic matter. Int. J. Environ. Res. 10, 465–470. 10.22059/ijer.2016.58766

[B106] Sayler G. S. Ripp S. (2000). Field applications of genetically engineered microorganisms for bioremediation processes. Curr. Opin. Biotechnol. 11, 286–289. 10.1016/S0958-1669(00)00097-5 10851144

[B107] Shahid A. Malik S. Zhu H. Xu J. Nawaz M. Z. Nawaz S. (2020). Cultivating microalgae in wastewater for biomass production, pollutant removal, and atmospheric carbon mitigation; a review. Sci. Total Environ. 704, 135303. 10.1016/j.scitotenv.2019.135303 31818584

[B108] Shaikh S. S. Abu-Dieyeh M. H. Al Naemi F. A. Ahmed T. Al-Ghouti M. A. (2020). Environmental impact of utilization of “produced water” from oil and gas operations in turfgrass systems. Sci. Rep. 10 (1), 15051. 10.1038/s41598-020-72069-5 32929117 PMC7490388

[B109] Shin S.-E. Lim J.-M. Koh H. G. Kim E. K. Kang N. K. Jeon S. (2016). CRISPR/Cas9-induced knockout and knock-in mutations in Chlamydomonas reinhardtii. Sci. Rep. 6, 27810. 10.1038/srep27810 27291619 PMC4904240

[B110] Shokravi Z. Shokravi H. Chyuan O. H. Lau W. J. Koloor S. S. R. Petrů M. (2020). Improving ‘lipid productivity’ in microalgae by bilateral enhancement of biomass and lipid contents: A review. Sustain. Switz. 12 (21), 9083. 10.3390/su12219083

[B111] Singh A. Ummalyma S. B. Sahoo D. (2020). Bioremediation and biomass production of microalgae cultivation in river water contaminated with pharmaceutical effluent. Bioresour. Technol. 307, 123233. 10.1016/j.biortech.2020.123233 32240927

[B112] Singh J. S. Abhilash P. C. Singh H. B. Singh R. P. Singh D. P. (2011). Genetically engineered bacteria: An emerging tool for environmental remediation and future research perspectives. Gene 480, 1–9. 10.1016/j.gene.2011.03.001 21402131

[B113] Sizova I. Greiner A. Awasthi M. Kateriya S. Hegemann P. (2013). Nuclear gene targeting in Chlamydomonas using engineered zinc-finger nucleases. Plant J. 73, 873–882. 10.1111/tpj.12066 23137232

[B114] Slattery S. S. Diamond A. Wang H. Therrien J. A. Lant J. T. Jazey T. (2018). An expanded plasmid-based genetic toolbox enables Cas9 genome editing and stable maintenance of synthetic pathways in *Phaeodactylum tricornutum* . ACS Synth. Biol. 7, 328–338. 10.1021/acssynbio.7b00191 29298053

[B115] Song I. Kim S. Kim J. Oh H. Jang J. Jeong S. J. (2022). Macular pigment-enriched oil production from genome-edited microalgae. Microb. Cell Fact. 21, 27. 10.1186/s12934-021-01736-7 35183173 PMC8858528

[B116] Song R. Zhai Q. Sun L. Huang E. Zhang Y. Zhu Y. (2019). CRISPR/Cas9 genome editing technology in filamentous fungi: Progress and perspective. Appl. Microbiol. Biotechnol. 103, 6919–6932. 10.1007/s00253-019-10007-w 31332488 PMC6690858

[B117] Srimongkol P. Sangtanoo P. Songserm P. Watsuntorn W. Karnchanatat A. (2022). Microalgae-based wastewater treatment for developing economic and environmental sustainability: Current status and future prospects. Front. Bioeng. Biotechnol. 10, 904046. 10.3389/fbioe.2022.904046 36159694 PMC9489850

[B118] Sriprang R. Hayashi M. Ono H. Takagi M. Hirata K. Murooka Y. (2003). Enhanced accumulation of Cd2+ by a Mesorhizobium sp. transformed with a gene from *Arabidopsis thaliana* coding for phytochelatin synthase. Appl. Environ. Microbiol. 69, 1791–1796. 10.1128/AEM.69.3.1791-1796.2003 12620871 PMC150072

[B119] Sukla L. B. Subudhi E. Pradhan D. (2019). The role of microalgae in wastewater treatment. Germany: Springer.

[B120] Sullivan Graham E. J. Dean C. A. Yoshida T. M. Twary S. N. Teshima M. Alvarez M. A. (2017). Oil and gas produced water as a growth medium for microalgae cultivation: A review and feasibility analysis. Algal Res. 24, 492–504. 10.1016/j.algal.2017.01.009

[B121] Takahashi K. Ide Y. Hayakawa J. Yoshimitsu Y. Fukuhara I. Abe J. (2018). Lipid productivity in TALEN-induced starchless mutants of the unicellular green alga Coccomyxa sp. strain Obi. Algal Res. 32, 300–307. 10.1016/j.algal.2018.04.020

[B122] Tran N. T. Kaldenhoff R. (2020). Achievements and challenges of genetic engineering of the model green alga Chlamydomonas reinhardtii. Algal Res. 50, 101986. 10.1016/j.algal.2020.101986

[B123] Varaprasad D. Narasimham D. Paramesh K. Sudha N. R. Himabindu Y. Keerthi Kumari M. (2021). Improvement of ethanol production using green alga Chlorococcum minutum. Environ. Technol. 42, 1383–1391. 10.1080/09593330.2019.1669719 31526318

[B124] Veiga M. C. Fontoura M. M. de Oliveira M. G. Costa J. A. V. Santos L. O. (2020). Magnetic fields: Biomass potential of spirulina sp. for food supplement. Bioprocess Biosyst. Eng. 43, 1231–1240. 10.1007/s00449-020-02318-4 32144594

[B125] Wang Q. Gong Y. He Y. Xin Y. Lv N. Du X. (2021). Genome engineering of *Nannochloropsis* with hundred‐kilobase fragment deletions by Cas9 cleavages. Plant J. 106, 1148–1162. 10.1111/tpj.15227 33719095

[B126] Wang X.-W. Huang L. Ji P.-Y. Chen C.-P. Li X.-S. Gao Y.-H. (2019). Using a mixture of wastewater and seawater as the growth medium for wastewater treatment and lipid production by the marine diatom Phaeodactylum tricornutum. Bioresour. Technol. 289, 121681. 10.1016/j.biortech.2019.121681 31247531

[B127] Watanabe M. M. Isdepsky A. (2021). Biocrude oil production by integrating microalgae polyculture and wastewater treatment: Novel proposal on the use of deep water-depth polyculture of mixotrophic microalgae. Energies 14, 6992. 10.3390/en14216992

[B128] Xu J. Soni V. Chopra M. Chan O. (2020a). Genetic modification of the HSP90 gene using CRISPR-cas9 to enhance thermotolerance in T. Suecica. Undergrad. Res. Nat. Clin. Sci. Technol. (URNCST) J. 4, 1–6. 10.26685/urncst.178

[B129] Xu M. Weng Q. Ji J. (2020b). Applications and advances of CRISPR/Cas9 in animal cancer model. Briefings Funct. Genomics 19, 235–241. 10.1093/bfgp/elaa002 32124927

[B130] Zhang S. Guo F. Yan W. Dai Z. Dong W. Zhou J. (2020). Recent advances of CRISPR/Cas9-Based genetic engineering and transcriptional regulation in industrial biology. Front. Bioeng. Biotechnol. 7, 459. 10.3389/fbioe.2019.00459 32047743 PMC6997136

[B131] Zhang X.-H. Tee L. Y. Wang X.-G. Huang Q.-S. Yang S.-H. (2015). Off-target effects in CRISPR/Cas9-mediated genome engineering. Mol. Ther. Nucleic Acids 4, e264. 10.1038/mtna.2015.37 26575098 PMC4877446

[B132] Zhang Y. Huang H. Zhang B. Lin S. (2016). TALEN- and CRISPR-enhanced DNA homologous recombination for gene editing in zebrafish. Methods Cell Biol. 135, 107–120. 10.1016/bs.mcb.2016.03.005 27443922

[B133] Zhang Y. Zhao L. Wang Y. Yang B. Chen S. (2008). Enhancement of heavy metal accumulation by tissue specific co-expression of iaaM and ACC deaminase genes in plants. Chemosphere 72, 564–571. 10.1016/j.chemosphere.2008.03.043 18471863

[B134] Zhu L. D. Li Z. H. Hiltunen E. (2016). Strategies for lipid production improvement in microalgae as a biodiesel feedstock. BioMed Res. Int. 2016, 1–8. 10.1155/2016/8792548 PMC5048031 27725942

